# Supplementary material for: An ESCRT-dependent step in fatty acid transfer from lipid droplets to mitochondria through VPS13D−TSG101 interactions
Source: Nat Commun. 2021 Feb 23;12:1252. doi: 10.1038/s41467-021-21525-5 (PMC7902631; doi:10.1038/s41467-021-21525-5)
Supplement: Supplementary file 1 — Supplementary information [file 41467_2021_21525_MOESM1_ESM.pdf]

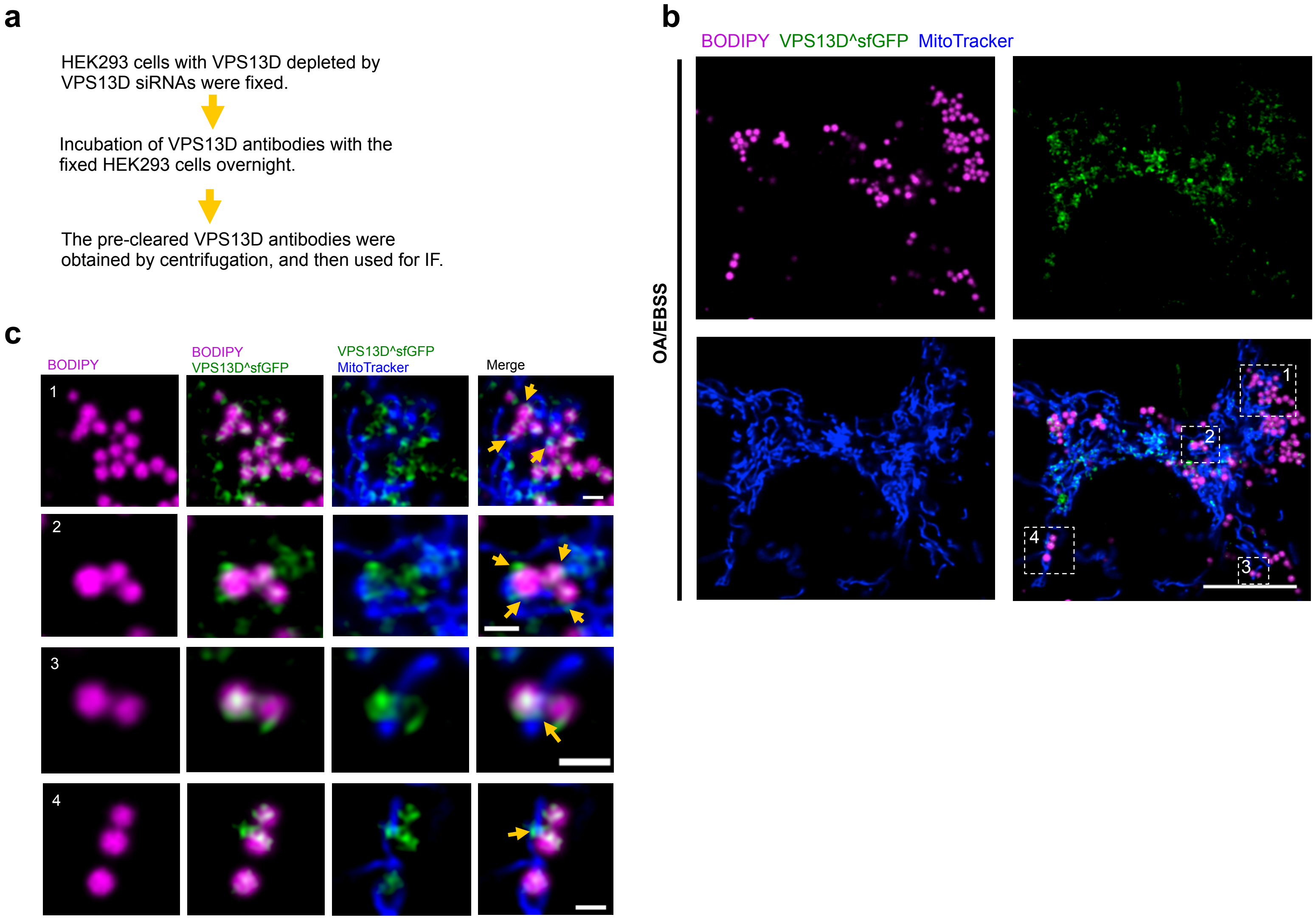
a
HEK293 cells with VPS13D depleted by VPS13D siRNAs were fixed.
Incubation of VPS13D antibodies with the fixed HEK293 cells overnight.
The pre-cleared VPS13D antibodies were obtained by centrifugation, and then used for IF.
b
BODIPY VPS13D^sfGFP MitoTracker
OA/EBSS
1
2
3
4
c
BODIPY
BODIPY
VPS13D^sfGFP
VPS13D^sfGFP
MitoTracker
Merge
1
2
3
4

Supplementary Fig. 1. Supplementary data to Fig. 1. a Workflow of pre-clearing VPS13D antibodies. **b** High-resolution confocal image of HEK293 cells expressing VPS13D^sfGFP (green), and labeled with MitoTracker Deep red (blue), and LDs marker BODIPY 558/568 (magenta) under OA/EBSS stimulation. **c** Four insets from boxed regions in (**b**). Yellow arrows denoted VPS13D^sfGFP puncta at mitochondria-LDs junctions. Scale bar, 10 μm in whole cell image in (**b**); 1 μm in (**c**).

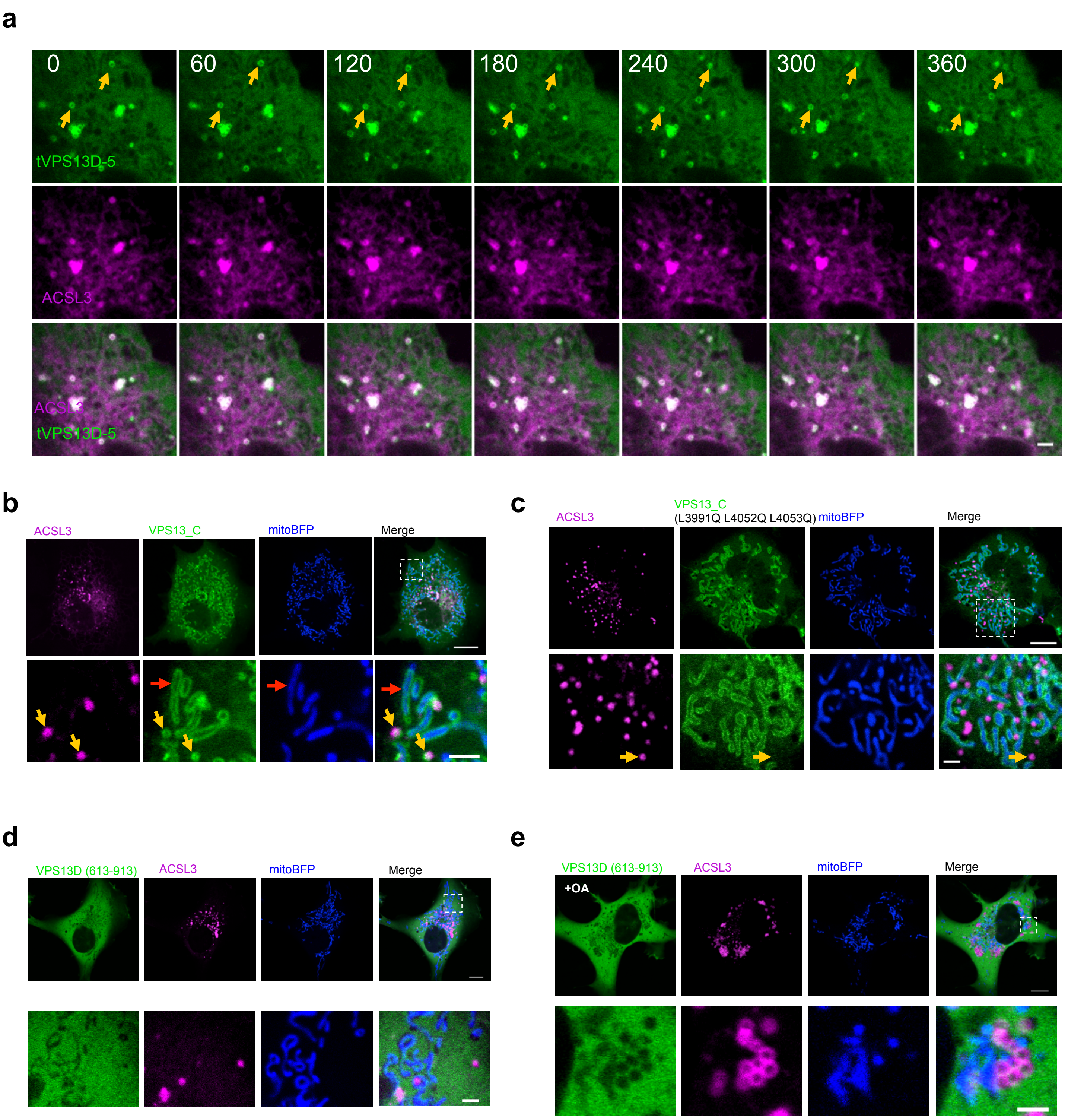
a
0
60
120
180
240
300
360
tVPS13D-5
ACSL3
ACSL3
tVPS13D-5
b
ACSL3
VPS13_C
mitoBFP
Merge
c
ACSL3
VPS13_C
(L3991Q L4052Q L4053Q)
mitoBFP
Merge
d
VPS13D (613-913)
ACSL3
mitoBFP
Merge
e
VPS13D (613-913)
ACSL3
mitoBFP
Merge
+OA

Supplementary Fig. 2. Supplementary data to Fig.2 and Fig. 3. a As in **Fig.2b**, time-lapse images of a COS7 cell expressing GFP-tVPS13D-5 and ACSL3-Halo with yellow arrows indicating GFP-tVPS13D-5 associated with LDs over time. Time in sec. **b** Confocal image of a COS7 cell expressing GFP-VPS13_C (green) and ACSL3-Halo (magenta) in CM without OA. Yellow arrows indicated the LDs associating with VPS13_C while red arrows denoted the mitochondria with VPS13_C. **c** Confocal image of a COS7 cell expressing GFP-VPS13_C (green) with three point mutations in the hydrophobic face of the two predicted amphipathic helices (L3991Q in helix-1; L4052Q, L4053Q in helix-2) in CM without OA. Yellow arrows indicated LDs without VPS13_C. **d**, **e** Confocal image of a COS7 cell expressing VPS13D (613-913)-GFP in CM without OA (**d**) or with OA (**e**). Top: whole cell image; Bottom: one inset from a boxed region in whole cell image. Scale bar, 2 μm in (**a**); 10 μm in whole cell image and 2 μm in insets in (**b**, **c**, **d** & **e**).

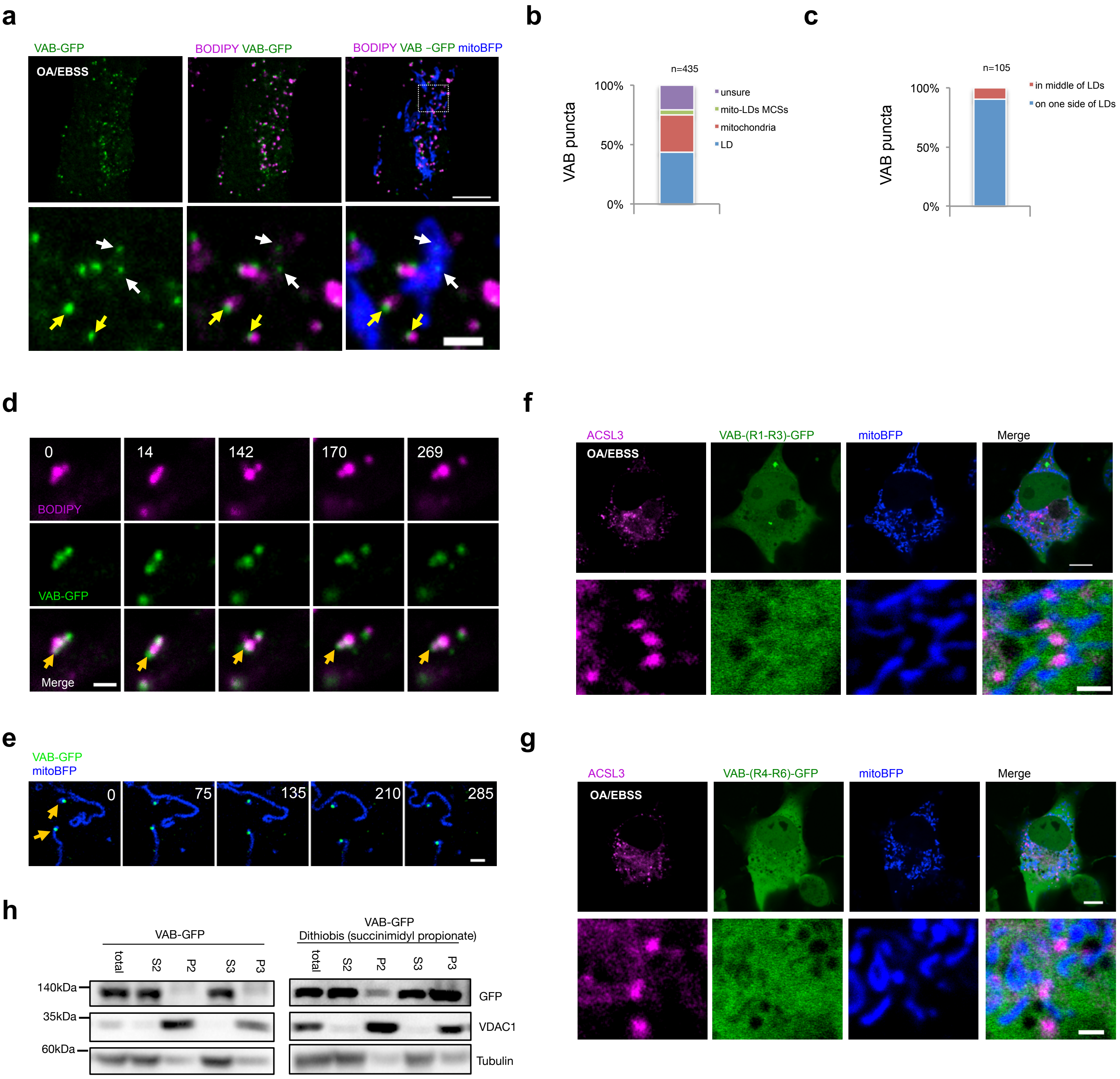
a
VAB-GFP
BODIPY VAB-GFP
BODIPY VAB -GFP mitoBFP
OA/EBSS
b
n=435
VAB puncta
100%
50%
0%
unsure
mito-LDs MCSs
mitochondria
LD
c
n=105
VAB puncta
100%
50%
0%
in middle of LDs
on one side of LDs
d
0
14
142
170
269
BODIPY
VAB-GFP
Merge
e
VAB-GFP
mitoBFP
0
75
135
210
285
f
ACSL3
VAB-(R1-R3)-GFP
mitoBFP
Merge
OA/EBSS
g
ACSL3
VAB-(R4-R6)-GFP
mitoBFP
Merge
OA/EBSS
h
VAB-GFP
VAB-GFP
Dithiobis (succinimidyl propionate)
total
S2
P2
S3
P3
140kDa
35kDa
60kDa
GFP
VDAC1
Tubulin

Supplementary Fig. 3. VAB domain of VPS13D associated with LDs or mitochondria. a Confocal image of a BODIPY 558/568-labeled HEK293 cell expressing VAB-GFP, mitoBFP with yellow arrows denoting VAB decorated LDs while white arrows indicating VAB decorated mitochondria. **b** Cellular distribution of VAB puncta (n=1849 from 17 cells). **c** Percentage of VAB puncta on one side of LDs (n=105 from 7 cells). At least three independent assays were performed with similar results in (**b**, **c**). **d**, **e** Time-lapse imaging of a region from a cell as in (**a**) with yellow arrows denoting VAB puncta stably associated with LDs (**d**) or mitochondria (**e**) over time. Time in sec. **f**, **g** Confocal image of a HEK293 cell expressing VAB-GFP (R1-R3) (**f**) or VAB-GFP (R4-R6) (**g**) along with ACSL3-mCherry and mitoBFP. **h** As in Fig. 3**d**, western blots demonstrations of the distribution of VAB-GFP in subcellular fractions. Anti-GFP, VDAC1 and Tubulin antibody was used. Left: cells were not Dithiobis (succinimidyl propionate) crosslinked; Right: cells were crosslinked prior to lysis. Scale bar, 10 μm in whole cell image and 2 μm in insets in **(a, f, g)**; 2 μm in (**d**, **e**).

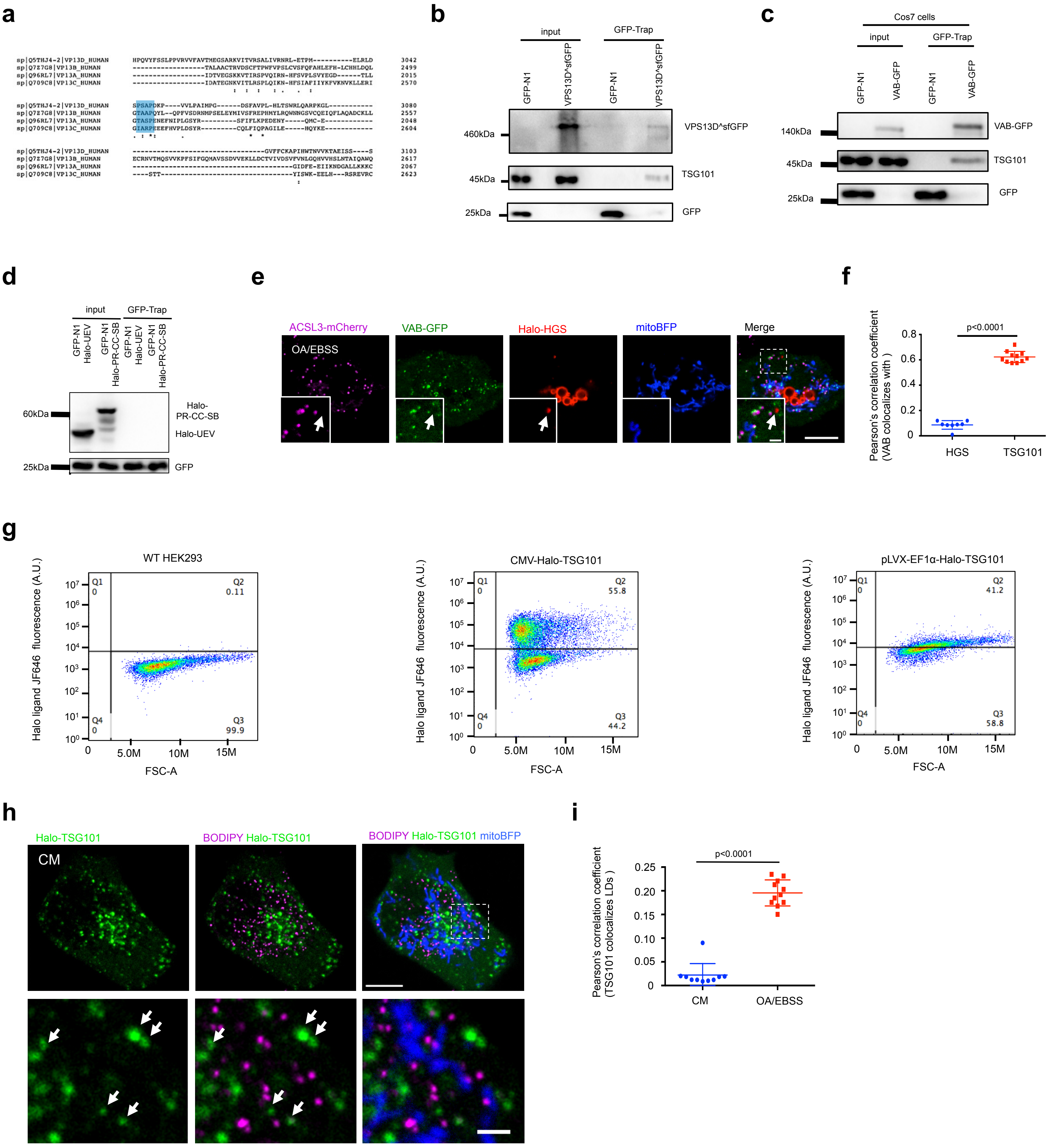
a
sp|Q5THJ4-2|VP13D_HUMAN
sp|Q7Z7G8|VP13B_HUMAN
sp|Q96RL7|VP13A_HUMAN
sp|Q709C8|VP13C_HUMAN
b
input
GFP-Trap
GFP-N1
VPS13D^sfGFP
460kDa
45kDa
25kDa
VPS13D^sfGFP
TSG101
GFP
c
Cos7 cells
input
GFP-Trap
GFP-N1
VAB-GFP
140kDa
45kDa
25kDa
VAB-GFP
TSG101
GFP
d
input
GFP-Trap
GFP-N1
Halo-UEV
Halo-PR-CC-SB
60kDa
25kDa
Halo-PR-CC-SB
Halo-UEV
GFP
e
ACSL3-mCherry
VAB-GFP
Halo-HGS
mitoBFP
Merge
OA/EBSS
f
Pearson's correlation coefficient (VAB colocalizes with)
p<0.0001
HGS
TSG101
g
WT HEK293
CMV-Halo-TSG101
pLVX-EF1α-Halo-TSG101
Halo ligand JF646 fluorescence (A.U.)
FSC-A
Q1 0
Q2 0.11
Q4 0
Q3 99.9
Q2 55.8
Q3 44.2
Q2 41.2
Q3 58.8
5.0M
10M
15M
h
Halo-TSG101
BODIPY Halo-TSG101
BODIPY Halo-TSG101 mitoBFP
CM
i
Pearson's correlation coefficient (TSG101 colocalizes LDs)
p<0.0001
CM
OA/EBSS

Supplementary Fig. 4. Supplementary data to Fig. 4. a Alignment of VAB domains among four human VPS13 proteins demonstrated that PSAP motif (highlighted by blue shadow) exclusively existed in VPS13D. **b** GFP-Trap assay of HEK293 cells expressing VPS13D^sfGFP showing a interaction between VPS13D^sfGFP and TSG101 with anti-GFP and TSG101 antibody. **c** GFP-Trap assay of COS7 cells stably expressing VAB-GFP indicating a interaction between VAB-GFP and endogenous TSG101 with anti-GFP and TSG101 antibody. **d** As a control to **Fig.4e**, GFP-Trap assay of COS7 cells expressing GFP-N1 (empty vector) along with either Halo-UEV or Halo-PR-CC-SB demonstrating that neither Halo-UEV nor Halo-PR-CC-SB interacted with GFP alone with anti-GFP and anti-Halo antibody. **e** Confocal image of a HEK293 cell expressing Halo-HGS (red), VAB-GFP (green), ACSL3-mCherry (magenta) and mitoBFP (blue) with white arrows denoting HGS not co-localizing with the VAB-GFP. **f** Relative co-localization between VAB-GFP and Halo-HGS or Halo-TSG101 upon OA/EBSS stimulations by Pearson's correlation coefficient analysis (n=11 cells). Three independent assays were performed with similar results. $p<0.0001$, two-tailed unpaired student's *t*-test. Mean ± SD. **g** Assessment of the expression level of Halo-TSG101 in CMV promoter versus EF1α promoter mediated ectopic expression by flow cytometry. A.U., arbitrary unit. FSC-A ,forward scatter area. **h** Confocal image of a BODIPY 558/568-labeled HEK293 cell expressing Halo-TSG101, mitoBFP with white arrows denoting TSG101 puncta not associating with LDs in CM. **i** Relative co-localization of Halo-TSG101 and LDs under normal conditions (n=10 cells) or OA/EBSS stimulations (n=11 cells) by Pearson's correlation coefficient analysis. Three independent assays were performed with similar results. p<0.0001, two-tailed unpaired student's *t*-test. Scale bar, 10 μm in whole cell image and 2 μm in insets in **(e, h)**.

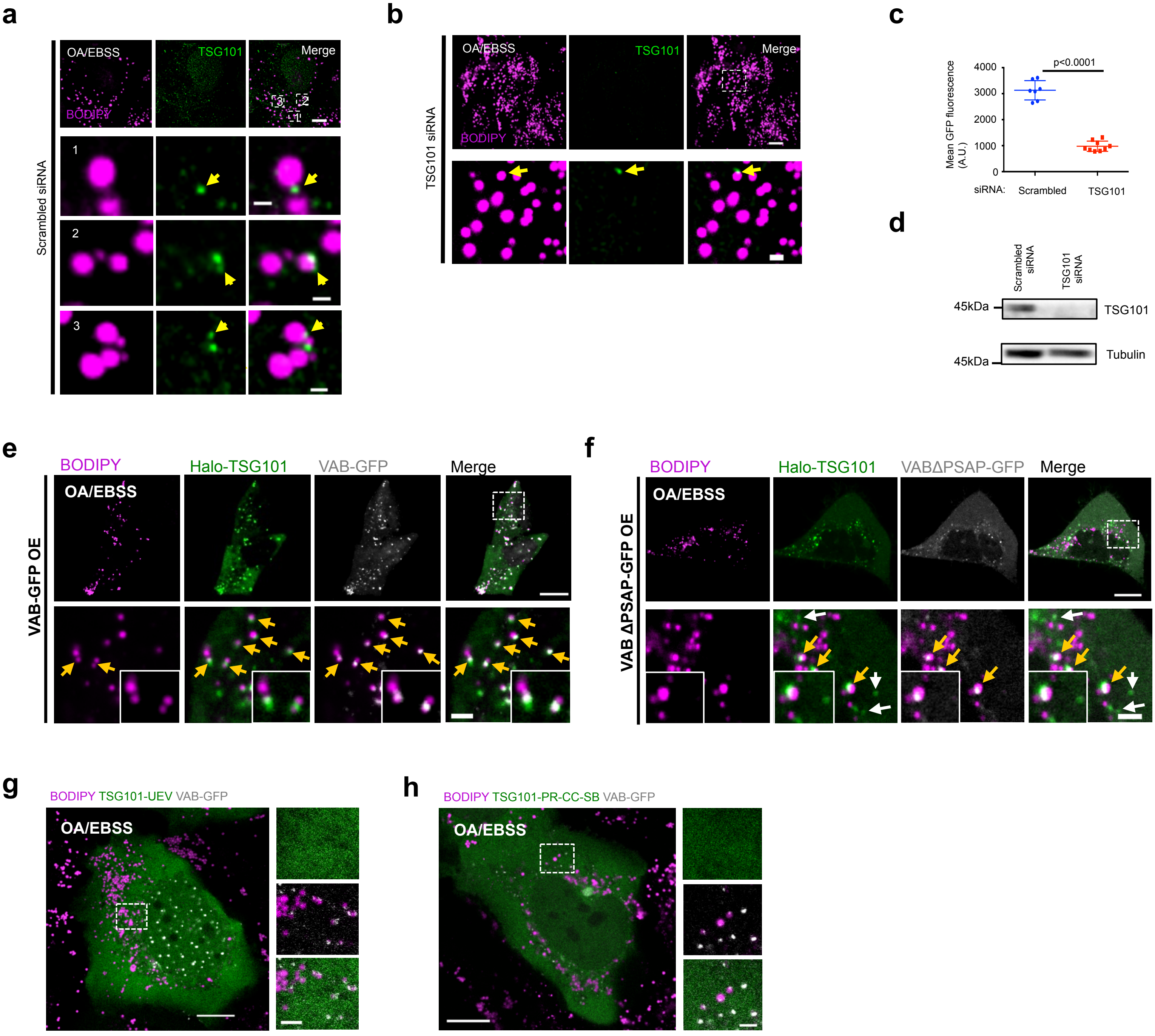

a
OA/EBSS
BODIPY
TSG101
Merge
Scrambled siRNA
1
2
3
b
OA/EBSS
BODIPY
TSG101
Merge
TSG101 siRNA
c
p<0.0001
Mean GFP fluorescence (A.U.)
4000
3000
2000
1000
0
siRNA:
Scrambled
TSG101
d
Scrambled siRNA
TSG101 siRNA
45kDa
TSG101
45kDa
Tubulin
e
BODIPY
Halo-TSG101
VAB-GFP
Merge
OA/EBSS
VAB-GFP OE
f
BODIPY
Halo-TSG101
VABΔPSAP-GFP
Merge
OA/EBSS
VAB ΔPSAP-GFP OE
g
BODIPY TSG101-UEV VAB-GFP
OA/EBSS
h
BODIPY TSG101-PR-CC-SB VAB-GFP
OA/EBSS

Supplementary Fig. 5. A fraction of endogenous TSG101 was localized to LDs under OA/EBSS stimulation. **a**, **b** IF of BODIPY 558/568-labeled HEK293 cells upon treatments with scrambled (**a**) or TSG101 siRNAs (**b**) using anti-TSG101 antibody with yellow arrows denoting TSG101 decorated LDs. **c** Quantification of GFP fluorescence intensity in either scrambled (n=35) or TSG101 siRNAs treated cells (n=43). A.U., arbitrary unit. Three independent assays were performed with similar results. *p*<0.0001, two-tailed unpaired student's *t*-test. Mean ± SD. **d** Western blots demonstrating the knockdown efficiency of TSG101 using anti-TSG101, anti-tubulin antibody. **e, f** Confocal image of BODIPY 558/568-labeled HEK293 cells expressing Halo-TSG101 (green) and either VAB-GFP (gray, **e**) or VAB-ΔPSAP-GFP (gray, **f**) in response to OA/EBSS stimulation. Top: whole cell image; Bottom: one inset from a boxed region in whole cell image with yellow arrows denoting TSG101 decorated LDs while white arrows indicating TSG101 puncta not on LDs. **g, h** As in (**e**, **f**), Confocal image of BODIPY 558/568-labeled HEK293 cells expressing VAB-GFP (gray) and either Halo-TSG101-UEV (green, **g**) or Halo-TSG101-PR-CC-SB (green, **h**) under OA/EBSS stimulation. Scale bar, 10 μm in whole cell image and 2 μm in insets in **(a, b, e, f, g & h)**.

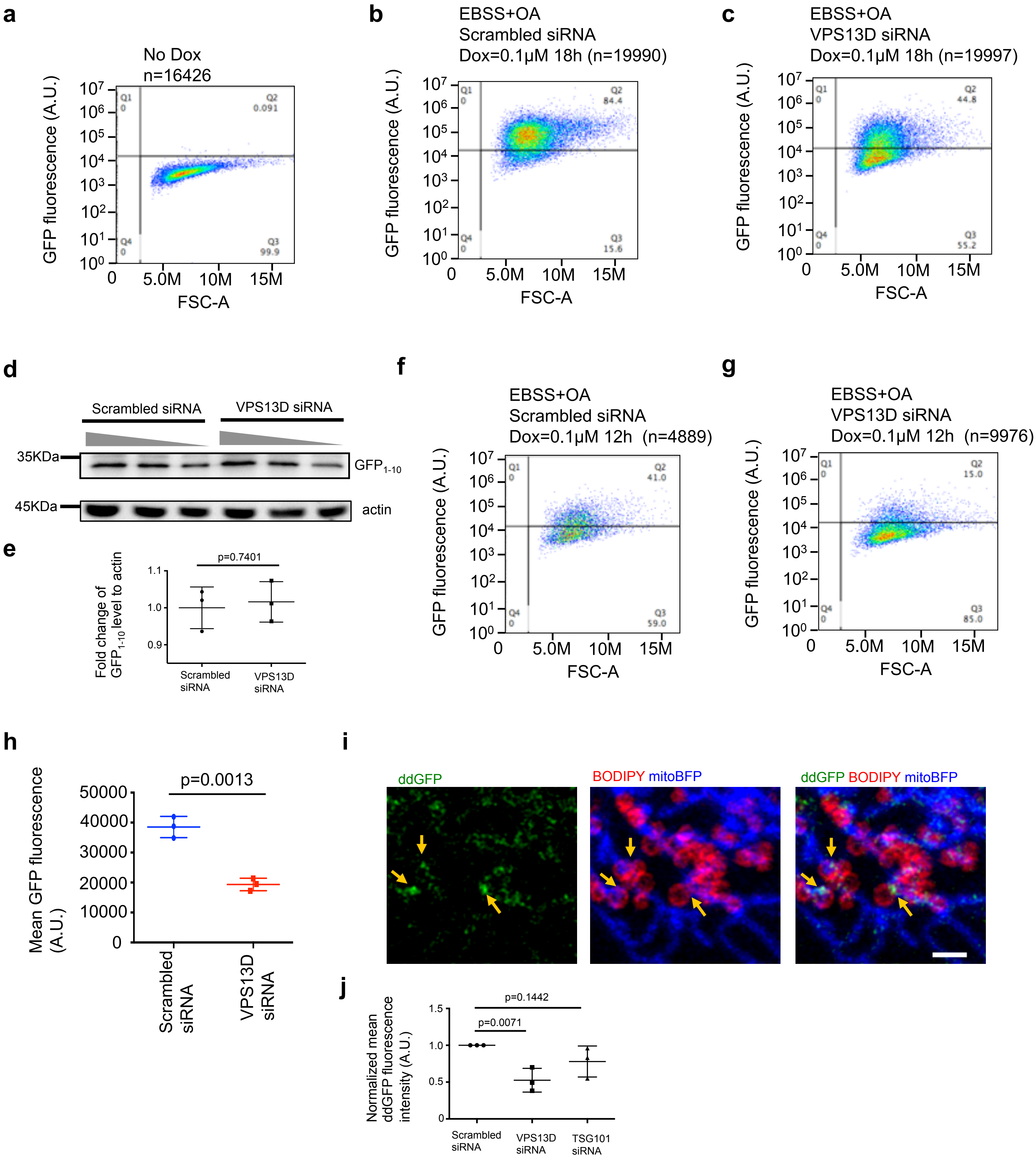

a
No Dox
n=16426
GFP fluorescence (A.U.)
FSC-A
b
EBSS+OA
Scrambled siRNA
Dox=0.1μM 18h (n=19990)
c
EBSS+OA
VPS13D siRNA
Dox=0.1μM 18h (n=19997)
d
Scrambled siRNA
VPS13D siRNA
35KDa
GFP1-10
45KDa
actin
e
p=0.7401
Fold change of GFP1-10 level to actin
Scrambled siRNA
VPS13D siRNA
f
EBSS+OA
Scrambled siRNA
Dox=0.1μM 12h (n=4889)
g
EBSS+OA
VPS13D siRNA
Dox=0.1μM 12h (n=9976)
h
p=0.0013
Mean GFP fluorescence (A.U.)
Scrambled siRNA
VPS13D siRNA
i
ddGFP
BODIPY mitoBFP
ddGFP BODIPY mitoBFP
j
p=0.1442
p=0.0071
Normalized mean ddGFP fluorescence intensity (A.U.)
Scrambled siRNA
VPS13D siRNA
TSG101 siRNA

Supplementary Fig. 6. Supplementary data to Fig. 5. a-c Measurement of mean GFP fluorescence intensity of mitochondria-LDs MCSs reporter cells by flow cytometry: no Dox addition (**a**, n=16426); scrambled (**b**, n=19998) or VPS13D siRNAs (**c**, n=19997) treated cells upon Dox induction (0.1μM, 18h). A.U., arbitrary unit. **d** Western blots showing the level of $GFP_{1\text{-}10}$ in scrambled or VPS13D siRNAs treated cells with varying amount of extract loaded (1.0x, 0.75x and 0.5x from left to right). **e** Fold change of $GFP_{1\text{-}10}$ to actin in scrambled or VPS13D siRNA treated mitochondria-LD reporter cells in **(d)**. Three biological replicates were performed. Two-tailed unpaired student's *t*-test. Mean ± SD. FSC-A, forward scatter area. **f, g** As in (**a-c**), measurement of mean GFP fluorescence intensity of mitochondria-LDs MCSs reporter cells with reduced Dox induction (0.1μM, 12h) by flow cytometry: scrambled (**f**, n=4889) or VPS13D siRNAs (**g**, n=9976) treated cells upon Dox induction. A.U., arbitrary unit. FSC-A, forward scatter area. **h** Quantification of mean GFP fluorescence of mitochondria-LDs contact sites reporter cells. A.U., arbitrary unit. Three independent assays were performed with similar results. Two-tailed unpaired student's *t*-test. Mean ± SD. **i** Representative image of the localization of reversible ddGFP (green) at mitochondria (blue)-LD (red) MCSs. Yellow arrow denoting ddGFP at such MCSs. ddGFP representing the fluorescence of dimerized ddGFP. **j** Measurement of mean ddGFP fluorescence by flow cytometry. A.U., arbitrary unit. Three independent experiments were analyzed with similar results. Two-tailed unpaired student's *t*-test. Mean ± SD. Scale bar, 2 μm in **(i)**.

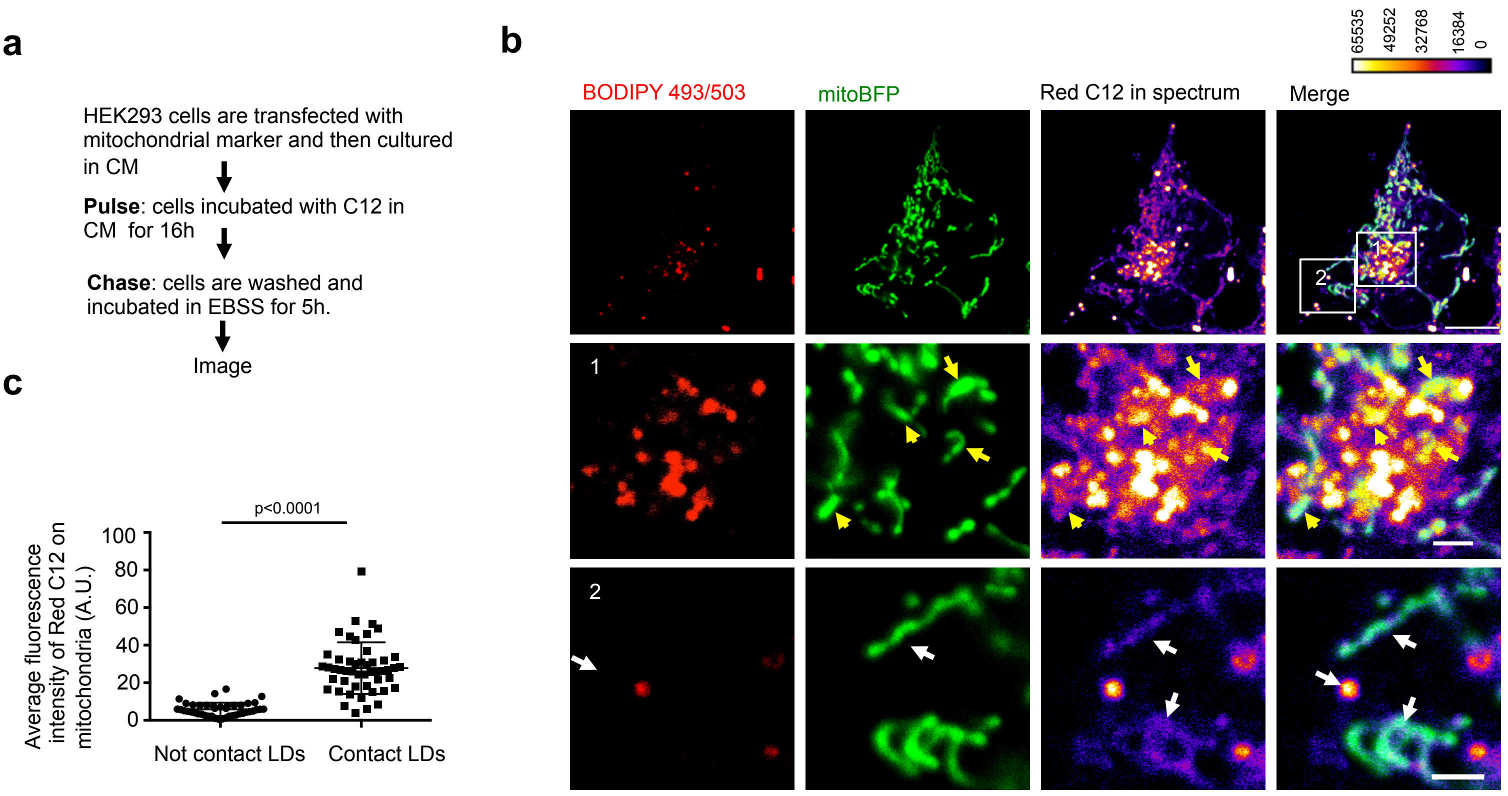

d

LTD Mutant: L42R, L66R, L85R, I148R, I152E, I174R, M181R, F203E, L278R, L293R, W319R

```
hATG2A   MSRWLWPWSNCVKERVCRYLLHHYLGHFFQEHLSLDQLSLDLYKGSVALRDIHLEIWSVN 60
hVP13D   -----------MLEGLVAWVLNTYLGKYV-NNLNTDQLSVALLKGAVELENLPLKKDALK 48
                    : :     :*   :. :. :::. .:*.: :  *   *.: *    :.

hATG2A   EVLESMESPLELVEGFVGSIEVAVPWAALLTDHCTVRVSGLQLTLQPRRGPAPGAADSQS 120
hVP13D   ----ELELPFEVKAGFIGKVTLQIPFYRPHVDPWVISISSLHLIGAPEKIQDFNDEKEKL 104
             . . *: .  * : .: : :*:     .   : :        .     .  .

hATG2A   WASCMTTSLQLAQ--ECL---RDGLPE-PSEPPQP-LEGLEM----FAQTIETVLRRIKV 169
hVP13D   LERERKKALLQALEEKWKNDRQQ--------------KGESYWYSVTASVVTRIVENIEL 150
                       .                      . .       .    :: .:::

hATG2A   TFLDTVVRVEHSPGDGERG-VAVEVRVQRLEYCDEAVRDPSQAPPVDV--HQPPAFLHKL 226
hVP13D   KIQDVHLRFEDGVTN-PSHPFAFGICIKNVSMQNAV----N------E--PVQKLMRKKQ 197
          : :  ::  .             :   .                         . :*

hATG2A   LQLAGVRLHYEELPAQE---------------------------------------EPPE 247
hVP13D   LDVAEFSIYWDVDCTLLGDL-P----QMELQEAM----------------AR--SMESRS 234
         : .    :  :

hATG2A   PPLQIG--SCSGYMELMVKLKQNEAFPGPKLEVAGQLGSLHLLLTPRQLQQLQ------- 298
hVP13D   HHYVLEPVFASALLKRNCSKKPLRSRHSPRIDCDIQLETIPLKLSQLQYRQIM------- 287
                             :        :.       : * :   :   :

hATG2A   -----------------------ELLSAVSLT---DHEGLADKLNKSRPLGA-----EDL 327
hVP13D   -----------------------EFLKELERKERQVK------FRRWKPKVAISKNCREW 318
                                ..*  :       :           *        :

hATG2A   WLIEQDLNQQLQA-----------------GAVAEPLSPDPLTNPLLNL--------D-- 360
hVP13D   WYFALNANLYEIREQRKRCTWDFMLHRARDAVSYTDKYFNKLKGGLLST--------DD- 369
         :                                     :
```

Supplementary Fig. 7. Supplementary data to Fig. 6. a Workflow of Red C12 pulse-cahse assays. **b** Confocal image of HEK293 cells labeled with mitoBFP (green), BODIPY 493/503 (red) and Red C12 (color spectrum) in response to 5 h of starvation. Top: whole cell image; Bottom: two inset from boxed region in whole cell image with yellow arrows denoting mitochondria adjacent to LDs while white arrows indicating mitochondria not contacting LDs. The scale for Color-coded Red C12 fluorescence was shown (0-65535, from completely dark to full brightness) on the top-right comer. **c** Measurement of the transfer level of Red C12 to mitochondria contacting LDs (n=43 mitochondria) versus mitochondria >2μm away from LDs during 2 min imaging (n=49 mitochondria) by measurements of the red C12 fluorescence in mitochondria. A.U., arbitrary unit. At least three independent assays were performed with similar results. p<0.0001, two-tailed unpaired student's t-test. Mean ± SD. **d** Alignment of human VPS13D-LTD (1-369 residues) with human ATG2A-LTD (1-360 residues). The conserved hydrophobic residues between VPS13D and ATG2A were mutated to hydrophilic residues, highlighted by blue shadow. Scale bar, 10 μm in whole cell image and 2 μm in insets in (**b)**.

Supplementary Table 1: DNA constructs used in this study.

Construct	Plasmid	Source
Halo-N1	mEGFP-N1(addgene#54767)	This study
Halo-C1	mEGFP-C1(addgene#54579)	This study
sfGFP-C1	mEGFP-C1	This study
VPS13D^sfGFP	sfGFP-C1	This study
GFP-tVPS13D-5	mEGFP-C1	This study
ACSL3-Halo	mEGFP-N1	This study
GFP-VPS13_C	mEGFP-C1	This study
GFP-VPS13_C mutant (L3991Q, L4052Q, L4053Q)	mEGFP-C1	This study
VPS13D^sfGFP (L3991Q, L4052Q, L4053Q)	VPS13D^sfGFP	This study
VPS13D (404-913)-GFP	mEGFP-N1	This study
VPS13D (613-913)-GFP	mEGFP-N1	This study
VAB-GFP (R1-R3)	mEGFP-N1	This study
VAB-GFP (R4-R6)	mEGFP-N1	This study
VPS13D (613-913)-GFP	mEGFP-N1	This study
VPS13D_N-GFP	mEGFP-N1	This study
GFP-VPS13D-ΔN	mEGFP-C1	This study
GFP-VPS13D-ΔN (L3991Q, L4052Q, L4053Q)	GFP-VPS13D-ΔN	This study
VAB-GFP	mEGFP-N1	This study

Halo-TSG101	mEGFP-N1	This study
VAB-ΔPSAP-GFP	mEGFP-N1	This study
Halo-UEV	mEGFP-C1	This study
Halo-PR-CC-SB	mEGFP-C1	This study
siRNA-resistant VPS13D^sfGFP	VPS13D^sfGFP	This study
siRNA resistant GFP-LTD-WT	mEGFP-C1	This study
siRNA resistant GFP-LTD-M	siRNA resistant GFP-LTD-WT	This study
pLVX-EF1α-VPS13D_N-GFP	pLVX-EF1a-acGFP-N1(Clontech, 631983)	This study
pLVX-EF1α-VAB-GFP	pLVX-EF1a-acGFP-N1	This study
pLVX-EF1α-Halo-TSG101	pLVX-EF1a-acGFP-N1	This study
pET28a-6xHis-SUMO		A gift from Hongjun Yu (Huazhong University of Science and Technology)
pET28a-6xHis-SUMO-VPS13D-LTD	pET28a-6xHis-SUMO	This study
Pcw57-Tom20-GFP$_{1\text{-}10}$-P2A-Plin2-3xFlag-7xGFP$_{11}$	pcw57-MCS1-P2A-MCS2(addgene#89180)	This study
Pcw57-flag-tom20-GB-P2A-plin2-3xflag-ddGA	pcw57-MCS1-P2A-MCS2	This study
Mito-BFP		Addgene#49151
psPAX2		Addgene#12260
pMD2. G		Addgene#12259
pEFIRES-P-ACSL3-mCherry		Addgene#87158
VPS13D-myc-flag		Origene#08156

Supplementary Table 2: DNA primers for generating plasmids and for qPCR assays in this study. The siRNA resistant VPS13D^sfGFP construct was generated by site-directed mutagenesis in which five silent mutations in siRNA seed region (GT AGC ACG TTT GAC ATG AA was mutated to GA AGT ACC TTC GAT ATG AA) were introduced in VPS13D^sfGFP, creating siRNA-resistant VPS13D^sfGFP mRNA that translates wild-type VPS13D^sfGFP. All of constructs used in this study were generated by using ClonExpress®II One Step Cloning kit (Vazyme, C112-01).

VPS13D^sfGFP

Name	Sequence
Fragement 1 forward	5'-GGGTATTCCCAAACACGGACCGATGAGCAAAGGAGAAGAACTTTTCACTG-3'
Fragement 1 reverse	5'-CGTTTTCCTCAGACTGCTAGCTTCGGTTACCGTGAAGGTTTTG-3'
Fragement 2 forward	5'-GCTAGCAGTCTGAGGAAAACGACAAGCACGGAGGAGCCCAG-3'
Fragement 2 reverse	5'-GCGTACGCGTttaGGAGTCCAGCTCCAGCTGCTCTCGG-3'
Fragement 3 forward	5'-GCTGGACTCCTAAACGCGTACGCGGCCGCTCGAGCAGAAACTC-3'
Fragement 3 reverse	5'-CATCGGTCCGTGTTTGGGAATACCCGTTGGGGAAG-3'

GFP-tVPS13D-5

Name	Sequence
forward	5'-TTATGATCTAGAGTCGCGGCCGCTCAGGAGTCCAGCTCCAGCTG-3'
reverse	5'-AGTCCGGACTCAGATCTCGAATGCACGACGCTTTCGAGC-3'

ACSL3-Halo

Name	Sequence
forward	5'-TCAGATCTCGAGCTCAAGCTTGCCACCTACCATGAATAACCACGTG TCTTCA-3'
reverse	5'-GTACCGTCGACTGCAGAATTGTTTTCTTCCATACATTCGCTCA-3'

GFP-VPS13_C

Name	Sequence
forward	5'-TACAAGTCCGGACTCAGATCTCCATTGGATCTTAAGGCCCTAA-3'
reverse	5'-CAGAATTCGAAGCTTGAGCTCTTATGCGTAGTTTATTTCTTGTGACAA-3'

GFP-VPS13_C mutant (L3991Q)

Name	Sequence
forward	5'-AAAGCACCcagGGGTTTCCTTTGATACGGTTTG-3'
reverse	5'-AAACCCctgGGTGCTTTTTAGGGCCTTAAGAT-3'

GFP-VPS13_C mutant (L3991Q, L4052Q, L4053Q)

Name	Sequence
forward	5'-TGGGGCAACAGAATGATGTTTCTGAAGGGGTTACTG-3'
reverse	5'-CATCATTCTGTTGCCCCATAGGATTGCCAAGAAA-3'

VPS13D (404-913)-GFP

Name	Sequence
forward	5'-TGAACCGTCAGATCCGCTAGCGCCACCATGGAGAGTCTGCGGGAGC C-3'
reverse	5'-CGGTGGATCCCGGGCCCGCGGAATCTGAGTGTCAGAAGTTTTCATTT C-3'

VPS13D (613-913)-GFP

Name	Sequence
forward	5'-TGAACCGTCAGATCCGCTAGCGCCACCATGTATGAGAGAAATCC-3'
reverse	5'-CGGTGGATCCCGGGCCCGCGGAATCTGAGTGTCAGAAGTTTTCATTT C-3'

VAB-GFP (R1-R3)

Name	Sequence
forward	5'-TGAACCGTCAGATCCGCTAGCGCCACCATGAAGCGCCGGCAGCCAT-3'
reverse	5'-CGGTGGATCCCGGGCCCGCGGCACCATATAGTTTTGGGTTCCAGG-3'

VAB-GFP (R4-R6)

Name	Sequence
forward	5'-TGAACCGTCAGATCCGCTAGCGCCACCATGTTAAAGATCTTCATTTCTGCTCCATAT-3'
reverse	5'-CGGTGGATCCCGGGCCCGCGGGATCTCAGAGGACCCTGCCC-3'

VPS13D_N-GFP

Name	Sequence
forward	5'-TGAACCGTCAGATCCGCTAGCGCCACCATGGAGAGTCTGCGGGAGCCTCA-3'
reverse	5'-CAGAATTCGAAGCTTGAGCTCATACAGCATCTCAAAAACGGGG-3'

GFP-VPS13D-ΔN

Name	Sequence
forward	5'-TCGAGCTCAAGCTTCGAATTCGCCACCATGTTGGAAGGCCTTGTAGCC-3'
reverse	5'-GGATCCCGGGCCCGCGGTACCTCACTTGTCGTCATCGTCTTTGTAGTCGGAGTCCAGCTCCAGCTGCTCTCGGTTTTC-3'

VAB-GFP

Name	Sequence
forward	5'-TCGAGCTCAAGCTTCGAATTCGCCACCATGGACCAGTATGTAAGTACCAAGGAATCG-3'
reverse	5'-CGGTGGATCCCGGGCCCGCGGCTGGAGTGCTCTAGTTGGTCCAT-3'

Halo-TSG101

Name	Sequence
forward	5'-ATTTCCCTCGAGCTCAAGCTTATGGCGGTGTCGGAGAGC-3'
reverse	5'-CGGGCCCGCGGTACCGTCGACTCAGTAGAGGTCACTGAGACCGG-3'

VAB-ΔPSAP-GFP

Name	Sequence
forward	5'-TGGATAGCGACAAGCCAGTGGTGCTTCCTGCT-3'
reverse	5'-TGGCTTGTCGCTATCCAGTCTTAGTTCCATTGGT-3'

Halo-UEV

Name	Sequence
forward	5'-ATTTCCCTCGAGCTCAAGCTTATGGCGGTGTCGGAGAGC-3'
reverse	5'-GTACCGTCGACTGCAGAATTCTCAAGGACGAGAGAAGACTGGAGGTT-3'

Halo-PR-CC-SB

Name	Sequence
forward	5'-ATTTCCCTCGAGCTCAAGCTTATTTCGGCATCCTATCCGCC-3'
reverse	5'-CGGGCCCGCGGTACCGTCGACTCAGTAGAGGTCACTGAGACCGG-3'

siRNA resistant GFP-LTD-WT

Name	Sequence
forward	5'-GGGAAGTACCTTCGATATGAATGGTTCTCTTGGCTGTTTA-3'
reverse	5'-TATCGAAGGTACTTCCCATTGAGACATTAACTTTGGTG-3'
Note	The DNA sequence of VPS13D-LTD-mut is synthesized by Genscript.

pLVX-EF1α-VPS13D_N-GFP

Name	Sequence
forward	5'-TCGAGCTCAAGCTTCGAATTCATGGAGAGTCTGCGGGAGCCTCA-3'
reverse	5'-CTCACCATGACCGGTGGATCATACAGCATCTCAAAAACGGGG-3'

pLVX-EF1α-VAB-GFP

Name	Sequence
forward	5'-TCGAGCTCAAGCTTCGAATTCGCCACCATGGACCAGTATGTAAGTACCAAGGAATCG-3'
reverse	5'-CTCACCATGACCGGTGGATCCTGGAGTGCTCTAGTTGGTCCAT-3'

pLVX-EF1α-Halo-TSG101

Name	Sequence
forward	5'-GGTACCGCGGGCCCGGGATCCGCCACCATGGAAATCGGTACT-3'
reverse	5'-GAATTATCTAGAGTCGCGGCCGCTCAGTAGAGGTCACTGAGACCGG-3'

Plin2-3xflag-7xGFP11

Name	Sequence
forward	5'-GGTCCTAGGCTGCAGACGCGTATGGCATCCGTTGCAGTTGA-3'
reverse	5'-CGCAACCCCAACCCCGGATCCATGAGTTTTATGCTCAGATC-3'

Plin2-3xflag

Name	Sequence
forward	5'-GGGAGACCCAAGCTGGCTAGCGCCACCATGGCATCCGTTGCAGTTGA-3'
reverse	5'-CTCACCATGGTACCGACCGGTCCTTTATCGTCATCATCCTTATAGTCA-3'

pET28a-6xHis-SUMO-VPS13D-LTD

Name	Sequence
forward	5'-CTGTACTTCCAATCCGCTAGCATGTTGGAAGGCCTTGTAGCC-3'
reverse	5'-TCCTTTCGGGCTTTGGTCGACtcaATACCACCATTCTCGGCAGTTC-3'

Human GAPDH qPCR

Name	Sequence
forward	5'-TCATTGACCTCAACTACATGGTTT-3'
reverse	5'-GAAGATGGTGATGGGATTTC-3'

Human VPS13D-1 qPCR

Name	Sequence

forward	5'- GTGACTGACAACAGATACGAGCCA-3'
reverse	5'- CGACCAACCCATGTAACCCA-3'

Human VPS13D-2 qPCR

Name	Sequence
forward	5'- TCGATATGCAGCACCAGATAAA-3'
reverse	5'- CTGGTGGGAGTGAAGAGAAATAA-3'

Human VPS13D-3 qPCR

Name	Sequence
forward	5'- GGATTTGCCTCAGATGGAGTTAC-3'
reverse	5'- TCCAGGACGTAGTGATGGCT-3'

Human VPS13A-1 qPCR

Name	Sequence
forward	5'- CGGTGCTATGGCTAAG -3'
reverse	5'- CCTCCACGAGTGATGC -3'

Human VPS13A-2 qPCR

Name	Sequence
forward	5'- TGGGCTTATGCTATACATGGCG -3'
reverse	5'- CCAAAGACACGAGAAGTTCACC -3'

Human VPS13A-3 qPCR

Name	Sequence
forward	5'- TGTGATGAATCGCCGATCTGA -3'
reverse	5'- CCATGTATAGCATAAGCCCACCA -3'

Supplementary Table 3: Oligonucleotides for siRNA used in this study.

human VPS13D

Name	Sequence
h-VPS13D-1	5’- GCAGAAGGATCCTTAAAGA-3’
h-VPS13D-2	5’-GTAGCACGTTTGACATGAA-3’
h-VPS13D-3	5’-CAAAGAGCCTCTACTATGA-3’

human TSG101

Name	Sequence
h-TSG101-1	5’- GAATCTGTATGCAGAAGAA -3’
h-TSG101-2	5’- GGTTACCCGTTTAGATCAA -3’
h-TSG101-3	5’- CCACAACAAGTTCTCAGTA -3’

human CHMP4B

Name	Sequence
h-CHMP4B-1	5’- GACGGCACATTATCAACCA -3’
h-CHMP4B-2	5’- GGACATCGATAAAGTTGAT -3’
h-CHMP4B-3	5’- GGCGGAATTAGAAGAACTA -3’

human CHMP1B

Name	Sequence
h-CHMP1B-1	5’- GGAGCAGGATGAACTGTCT -3’
h-CHMP1B-2	5’- GGCGGTGAATTTCTTGAGA -3’
h-CHMP1B-3	5’- CATGGAAGTTGCGAGGATA -3’

human CHMP6

Name	Sequence
h-CHMP6 -1	5’- TCACCCAGATCGAAATGAA -3’
h-CHMP6 -2	5’- TGCTCAAGAAGAAGCGATA -3’

h-CHMP6 -3	5’- GCTTCACTCAGGAGGATGA -3’

human IST1

Name	Sequence
h-IST1-1	5’- CCAGACCTGCAGATAACTA -3’
h-IST1-2	5’- GACATTAATGCTGATAAGA -3’
h-IST1-3	5’- AGAGACAGATCTTATTGAT-3’

human ALIX

Name	Sequence
h-ALIX -1	5’- GAAGGATGCTTTCGATAAA -3’
h-ALIX -2	5’- GGCACAGGCTCAAGAAGTA -3’
h-ALIX -3	5’- GAACCTGGATAATGATGAA-3’

scrambled siRNA

Name	Sequence
scrambled siRNA	5’-CGUUAAUCGCGUAUAAUACGCGUAT-3′
