# Supplementary material for: An ESCRT-dependent step in fatty acid transfer from lipid droplets to mitochondria through VPS13D−TSG101 interactions
Source: Nat Commun. 2021 Feb 23;12:1252. doi: 10.1038/s41467-021-21525-5 (PMC7902631; doi:10.1038/s41467-021-21525-5)
Supplement: Supplementary file 5 — Description of additional supplementary files [file 41467_2021_21525_MOESM5_ESM.docx]

Description of additional supplementary information

Title: Supplementary Movie 1

Description: Time-lapse images of a BODIPY-C12 (gray)-labeled HEK293 cell expressing Halo-TSG101 (green), VAB-GFP (red), mitoBFP (blue) showing that a LD contacted mitochondria with Halo-TSG101 and VAB-GFP at MCSs. Note that the size of LDs gradually decreased upon its contacts with mitochondria. Time intervals: 18 sec; Scar bar: 2 μm.

Title: Supplementary Movie 2

Description: Time-lapse images of a BODIPY 558/568 (magenta)- labeled HEK293 cell expressing mitoBFP (blue) showing the dynamic interactions between mitochondria and LDs in scrambled (top panel) or VPS13D siRNAs (bottom panel) treated cells. Left panel: mitochondria (blue) and LDs (magenta); right panel: merged image of mitochondria (blue), LDs (magenta) and co-localized pixels between mitochondria and LDs (white). Time intervals: 13.19 sec for scrambled siRNA treated cells and 19.28 sec for VPS13D siRNA treated cells; Scar bar: 10 μm.
