# Supplementary material for: An ESCRT-dependent step in fatty acid transfer from lipid droplets to mitochondria through VPS13D−TSG101 interactions
Source: Nat Commun. 2021 Feb 23;12:1252. doi: 10.1038/s41467-021-21525-5 (PMC7902631; doi:10.1038/s41467-021-21525-5)
Supplement: Supplementary file 6 — Reporting Summary [file 41467_2021_21525_MOESM6_ESM.pdf]

nature research

Corresponding author(s): Wei-Ke Ji

Last updated by author(s): Jan 24, 2021

Reporting Summary

Nature Research wishes to improve the reproducibility of the work that we publish. This form provides structure for consistency and transparency in reporting. For further information on Nature Research policies, see our Editorial Policies and the Editorial Policy Checklist.

Statistics

For all statistical analyses, confirm that the following items are present in the figure legend, table legend, main text, or Methods section.

n/a	Confirmed	
☐	☒	The exact sample size (*n*) for each experimental group/condition, given as a discrete number and unit of measurement
☐	☒	A statement on whether measurements were taken from distinct samples or whether the same sample was measured repeatedly
☐	☒	The statistical test(s) used AND whether they are one- or two-sided<br>*Only common tests should be described solely by name; describe more complex techniques in the Methods section.*
☒	☐	A description of all covariates tested
☐	☒	A description of any assumptions or corrections, such as tests of normality and adjustment for multiple comparisons
☐	☒	A full description of the statistical parameters including central tendency (e.g. means) or other basic estimates (e.g. regression coefficient) AND variation (e.g. standard deviation) or associated estimates of uncertainty (e.g. confidence intervals)
☐	☒	For null hypothesis testing, the test statistic (e.g. *F*, *t*, *r*) with confidence intervals, effect sizes, degrees of freedom and *P* value noted<br>*Give P values as exact values whenever suitable.*
☒	☐	For Bayesian analysis, information on the choice of priors and Markov chain Monte Carlo settings
☒	☐	For hierarchical and complex designs, identification of the appropriate level for tests and full reporting of outcomes
☒	☐	Estimates of effect sizes (e.g. Cohen's *d*, Pearson's *r*), indicating how they were calculated

Our web collection on statistics for biologists contains articles on many of the points above.

Software and code

Policy information about availability of computer code

Data collection	Images and Time-lapse movies were collected using ZEISS LSM780 laser scanning confocal microscope controlled by Zeiss Zen (version 2011) software and Leica SP8 equipped with Lightning super-resolution module controlled by LAS X software (version 4.0.2.22563).
Data analysis	The co-localized pixels between mitochondria and lipid droplets were analyzed using 'colocalization' plugin in ImageJ (2.0.0-rc-66/1.52b; NIH).<br>Fluorescent intensity was analyzed using imageJ software (2.0.0-rc-66/1.52b; NIH).<br>Statistical analyses were performed using GraphPad Prism 6.0 and Microsoft Excel (2011 and 2013).<br>The HeliQuest tool (v1.2; heliquest.ipmc.cnrs.fr) was used for the search of amphipathic helices in the VPS13_C domain.

For manuscripts utilizing custom algorithms or software that are central to the research but not yet described in published literature, software must be made available to editors and reviewers. We strongly encourage code deposition in a community repository (e.g. GitHub). See the Nature Research guidelines for submitting code & software for further information.

Data

Policy information about availability of data

All manuscripts must include a data availability statement. This statement should provide the following information, where applicable:

- Accession codes, unique identifiers, or web links for publicly available datasets
- A list of figures that have associated raw data
- A description of any restrictions on data availability

All the data and relevant materials, including reagents and primers, that support the findings of this study are available from the corresponding author upon reasonable request. The source data are provided as a file “Source Data”, and Source data are provided with this paper.

April 2020

Field-specific reporting

Please select the one below that is the best fit for your research. If you are not sure, read the appropriate sections before making your selection.

☒ Life sciences ☐ Behavioural & social sciences ☐ Ecological, evolutionary & environmental sciences

For a reference copy of the document with all sections, see nature.com/documents/nr-reporting-summary-flat.pdf

Life sciences study design

All studies must disclose on these points even when the disclosure is negative.

Sample size	All western blots, immunoprecipitation, seahorse assays, and in vitro protein-lipid binding assays were from at least three biological replicates and representative results are shown. Images from indirect IF of endogenous VPS13D using anti-VPS13D antibodies in HEK293 cells are representative from four independent experiments. Images from indirect IF of endogenous TSG101 using anti-TSG101 antibodies in HEK293 cells are representative from three independent experiments. Live imaging of transiently transfected cells expressing full length VPS13D^sfGFP was performed in HEK293 cells as VPS13D^sfGFP was too large to efficiently transfect other cell lines. Live imaging of transiently transfected cells expressing VPS13D mutants was performed in two cell lines: COS7 and HEK293. Cells expressing VPS13D^sfGFP or VPS13D mutants were imaged between 5 and 40 independent live-cell imaging sessions; cells expressing Halo-TSG101 and TSG101 mutants were imaged between 3 and 25 different sessions. Cells expressing VAPB-GFP and Halo-TSG101 were imaged at least 30 independent live-cell imaging sessions and repeated in three biological replicates in TEM with similar results. The splitGFP-based mito-LD MCSs reporter cells were imaged in at least four independent live-cell imaging sessions, and were applied to flow cytometry three times under two different conditions of Dox inductions with similar results. The ddGFP-based mito-LD MCSs reporter cells were imaged in three independent live-cell imaging sessions, and were applied to flow cytometry three times with similar results. HEK293 cells treated either with scrambled or VPS13D siRNAs were imaged between 3 and 10 independent live-cell imaging sessions for the quantifications of mitochondria-LD interactions. HEK293 cells treated either with scrambled, VPS13D, TSG101, CHMP4B, CHMP1B, CHMP6, IST1 or ALIX siRNAs were imaged in at least 4 independent live-cell imaging sessions. VPS13D-suppressed HEK293 cells rescued either with siRNA-resistant full length VPS13D^sfGFP, siRNA-resistant GFP-LTD or siRNA-resistant GFP-LTD Mutant were imaged in three independent live-cell imaging sessions.
Data exclusions	No data were excluded from the analyses.
Replication	All of experiments were performed at least three times. Experimental results are reliably reproduced.
Randomization	Not relevant to this study because cells/samples were analyzed in the same way.
Blinding	The investigators were not blinded during data collection. Blinding was used during analysis where the data were quantified/measured blind to the treatments.

Reporting for specific materials, systems and methods

We require information from authors about some types of materials, experimental systems and methods used in many studies. Here, indicate whether each material, system or method listed is relevant to your study. If you are not sure if a list item applies to your research, read the appropriate section before selecting a response.

Materials & experimental systems

n/a	Involved in the study	
☐	☒	Antibodies
☐	☒	Eukaryotic cell lines
☒	☐	Palaeontology and archaeology
☒	☐	Animals and other organisms
☒	☐	Human research participants
☒	☐	Clinical data
☒	☐	Dual use research of concern

Methods

n/a	Involved in the study	
☒	☐	ChIP-seq
☐	☒	Flow cytometry
☒	☐	MRI-based neuroimaging

Antibodies

Antibodies used	Anti-VPS13D (A304-691A, Bethy Laboratories. Inc), Anti-GFP (AE011, Abclonal), anti-Halo (G9211; Promega), anti-Tubulin (100109-MM05T; Sinobiological), anti-actin (20536-1-AP; Proteintech), anti-VDAC1 (55259-1-AP; Proteintech), anti-TSG101 (A1692; Abclonal) were used at 1:1000 dilutions for Western blots. Anti-VPS13D (A304-691A, Bethy Laboratories. Inc) and anti-TSG101 (SC7964; Santa Cruz Biotechnology) antibodies were used 1:100 for immunofluorescence (IF).
Validation	The VPS13D and TSG101 antibody were validated in IF by siRNA-meidated suppression by this study. (Figs. 1a-c and S5a-d). All commercial antibodies are wildly used common antibodies. Anti-GFP (AE011, Abclonal), anti-Halo (G9211; Promega), anti-Tubulin (100109-MM05T; Sinobiological), anti-actin (20536-1-AP; Proteintech), anti-VDAC1 (55259-1-AP; Proteintech) were validated by manufacturer and supported by multiple publications (31980630; 32345978; 31849517; 30568162; 31767755).

April 2020

Eukaryotic cell lines

Policy information about cell lines

Cell line source(s)	Human embryonic kidney 293 cells (ThermoFisher, R70507), African green monkey kidney fibroblast-like COS7 cell line (CRL-1651; ATCC), human liver cancer HepG2 cell line (HB-8065; ATCC) and Human embryonic kidney 293T cells (HEK293T/17; CRL-11268; ATCC) were grown in DMEM (Invitrogen) supplemented with 10% fetal bovine serum (Gibco) and 1% penicillin/streptomycin.
Authentication	All cell lines used in this study present the characteristic morphology. All cell lines were authenticated by the provider and were cultured according to the protocols from the providers, and no further authentication procedure was performed.
Mycoplasma contamination	All of the cell lines used in this study are free of mycoplasma contamination.
Commonly misidentified lines (See ICLAC register)	No commonly misidentified cell lines were used.

Flow Cytometry

Plots

Confirm that:

☒ The axis labels state the marker and fluorochrome used (e.g. CD4-FITC).

☒ The axis scales are clearly visible. Include numbers along axes only for bottom left plot of group (a 'group' is an analysis of identical markers).

☐ All plots are contour plots with outliers or pseudocolor plots.

☒ A numerical value for number of cells or percentage (with statistics) is provided.

Methodology

Sample preparation	Adherent mammalian cells were used in flow cytometry assays. Briefly, cells were trypsinized and washed with PBS for three times prior to FACS analysis.
Instrument	BD FACSAria III
Software	BD Accuri C6
Cell population abundance	All the samples analyzed by flow cytometry were either a same cell line with different treatments (Fig. S6a-c, f, g) or the same cells expressing different constructs (Fig. S4g). For each run, cells with a number of between 5000 and 20000 cells were analyzed.
Gating strategy	Untransfected, wild type cells were used as negative controls to set up a baseline, which is above approximate 99.9% of untransfected cells and is shown in each panel in Fig. S4g, and S6a-c, f, g. Transfected cell populations were determined based on the baseline.

☐ Tick this box to confirm that a figure exemplifying the gating strategy is provided in the Supplementary Information.
